# Antileukemic Efficacy of Continuous vs Discontinuous Dexamethasone in Murine Models of Acute Lymphoblastic Leukemia

**DOI:** 10.1371/journal.pone.0135134

**Published:** 2015-08-07

**Authors:** Laura B. Ramsey, Laura J. Janke, Monique A. Payton, Xiangjun Cai, Steven W. Paugh, Seth E. Karol, Landry Kamdem Kamdem, Cheng Cheng, Richard T. Williams, Sima Jeha, Ching-Hon Pui, William E. Evans, Mary V. Relling

**Affiliations:** 1 Pharmaceutical Sciences Department, St. Jude Children’s Research Hospital, Memphis, Tennessee, United States of America; 2 Department of Pathology, St. Jude Children’s Research Hospital, Memphis, Tennessee, United States of America; 3 Harding University College of Pharmacy, Searcy, Arkansas, United States of America; 4 Biostatistics Department, St. Jude Children’s Research Hospital, Memphis, Tennessee, United States of America; 5 Puma Biotechnology Inc., Los Angeles, California, United States of America; 6 Department of Oncology, St. Jude Children’s Research Hospital, Memphis, Tennessee, United States of America; University of Sydney, AUSTRALIA

## Abstract

Osteonecrosis is one of the most common, serious, toxicities resulting from the treatment of acute lymphoblastic leukemia. In recent years, pediatric acute lymphoblastic leukemia clinical trials have used discontinuous rather than continuous dosing of dexamethasone in an effort to reduce the incidence of osteonecrosis. However, it is not known whether discontinuous dosing would compromise antileukemic efficacy of glucocorticoids. Therefore, we tested the efficacy of discontinuous dexamethasone against continuous dexamethasone in murine models bearing human acute lymphoblastic leukemia xenografts (n = 8 patient samples) or murine BCR-ABL+ acute lymphoblastic leukemia. Plasma dexamethasone concentrations (7.9 to 212 nM) were similar to those achieved in children with acute lymphoblastic leukemia using conventional dosages. The median leukemia-free survival ranged from 16 to 59 days; dexamethasone prolonged survival from a median of 4 to 129 days in all seven dexamethasone-sensitive acute lymphoblastic leukemias. In the majority of cases (7 of 8 xenografts and the murine BCR-ABL model) we demonstrated equal efficacy of the two dexamethasone dosing regimens; whereas for one acute lymphoblastic leukemia sample, the discontinuous regimen yielded inferior antileukemic efficacy (log-rank p = 0.002). Our results support the clinical practice of using discontinuous rather than continuous dexamethasone dosing in patients with acute lymphoblastic leukemia.

## Introduction

With increasing intensity of treatment to improve the cure rates for acute lymphoblastic leukemia (ALL), toxic complications of chemotherapy have also increased. The incidence of osteonecrosis is high; 18% of patients experienced symptomatic osteonecrosis in one prospective study,[1] although others have reported rates between 1–25%.[2–8] Osteonecrosis is defined as bone death resulting from poor blood supply.[9] The development of osteonecrosis has been associated with the intensity of the drug exposure, type of steroid (dexamethasone > prednisone),[5] age, gender, asparaginase antibodies,[10] and genetics.[1, 11] Our group demonstrated that the initiating lesion in the mouse model of glucocorticoid-induced osteonecrosis is likely arteriopathy within the vessels supplying the distal femur.[12] Dexamethasone may contribute to osteonecrosis via its effects on lipids, coagulation, fibrinolysis, and direct toxic effects on vasculature and bone.[13, 14]

The Children’s Oncology Group trial CCG-1961 found that osteonecrosis was more common in the group receiving continuous dosing of dexamethasone versus those receiving discontinuous (alternate week) dexamethasone despite lower cumulative doses,[15, 16] with no reported loss of efficacy with discontinuous versus continuous dexamethasone when used in the context of a second delayed intensification phase. However, the efficacy assessment was complicated by the additional vincristine, anthracycline, and asparaginase given during an additional course of reinduction.[15, 16] Many ALL clinical trials now include a discontinuous glucocorticoid schedule, in which patients receive dexamethasone daily for 5–8 days, none for a period of some days, and then daily again, in an attempt to reduce the incidence of osteonecrosis, without evidence on whether discontinuous and continuous dexamethasone dosing have equivalent antileukemic effect.

Using a murine model of osteonecrosis, our group previously found that mice treated with a discontinuous regimen of dexamethasone developed less osteonecrosis than those treated with a continuous regimen,[17] consistent with clinical data in ALL [15, 16] and systemic lupus erythematosus patients.[18] Herein, we compared the antileukemic efficacy of these same dexamethasone regimens in a murine BCR-ABL *Arf* ^*-/-*^ preB cell ALL model in two different strains of mice [19] and in xenograft models of eight different primary ALL samples.

## Materials & Methods

### Murine leukemia cells

BCR-ABL (p185+) luciferase-positive cell lines were generated from bone marrow of *Arf* ^-/-^ mice on the C57Bl/6J and 129X1SvJ backgrounds as described previously.[19–21] Cells were thawed and recovered in culture for three days prior to intravenous tail vein injection of 2x10^3^ cells into non-irradiated male 8-week old matched syngenic mice (C57Bl/6 or 129X1SvJ, *Arf*-wildtype).

### Passage and transduction of patient leukemia cells

Bone marrow aspirates were cryopreserved at the time of ALL diagnosis by the St. Jude Tissue Resources Lab and kept at -140°C. Patient demographic details can be found in S1 Table. Written informed consent was obtained from patients or their parents/guardians, and assent from the patients, as appropriate. The consent, research and use of these samples were approved by the St. Jude Children’s Research Hospital Institutional Review Board on protocol XPD10-102. Patient samples were thawed, washed and injected via tail vein (1–5 million cells per mouse) into non-irradiated 8 week old female NOD. Cg-*Prkdc* ^*scid*^ *Il2rg* ^*tm1Wjl*^/SzJ (NSG) mice. The samples were passaged serially through NSG mice. Bone marrow isolated from the first passage was transduced with a lentiviral vector containing YFP and luciferase for the second passage, sorted for YFP+ cells prior to the third passage, and tested for *in vivo* efficacy of continuous and discontinuous dexamethasone dosing on the fourth passage.

When leukemia was evident in the blood of the first passage in mice by flow cytometry for hCD45 (see below), mice were sacrificed and bone marrow was isolated by flushing the femurs and tibias of each mouse with RPMI-1640 (Lonza BioWhittaker, Walkersville, MD) supplemented with 10% FBS (Thermo Scientific Hyclone, Logan, UT) and 2 mM L-glutamine (Lonza BioWhittaker, Walkersville, MD). The marrow was macerated over a sterile cell strainer (40 μm mesh) before counting cells with the Nexcelom Cellometer Vision, using the acridine orange propidium iodide (AOPI) assay (Nexcelom Bioscience LLC, Lawrence, MA). Non-tissue culture treated plates (Becton Dickinson Labware, Franklin Lakes, NJ) were coated with 20 ug/mL RetroNectin (Takara Bio Inc, Shiga, Japan) for 2 hours. Lentivirus encoding firefly luciferase and yellow fluorescent protein (CL20SF2-Luc2aYFP with VSV-G envelope, St. Jude Children’s Research Hospital Vector Lab, Memphis, TN) was mixed with bone marrow cells at a multiplicity of infection of 50 in IMDM + Glutamax media (GIBCO, Grand Island, NY) containing 20 ng/mL hIL-3 (Peprotech, Rocky Hill, NJ), 10 ng/mL hIL-7 (Peprotech), 50 ng/mL hSCF (Peprotech), 20 ng/mL hFLT3L (Peprotech), 2% human albumin (Sigma-Aldrich, St. Louis, MO), 1x ITS (10 mg/L insulin, 5.5 mg/L transferrin, 6.7 ug/L selenious acid, Cellgro, Manassas, VA), at a concentration of 1.13 x 10^6^ cells/mL. The plate was incubated at 37°C, and was swirled every 2 hours. The media was removed and replaced after 6 hours, and the plate incubated overnight at 37°C. The cells were dissociated from the retronectin with PBS + 0.02% EDTA, washed three times with PBS and reinjected into recipient NSG mice via tail vein.

At the second passage, when leukemia was evident in the blood by flow cytometry or by Xenogen imaging (see below), the mice were sacrificed and bone marrow harvested as above and sorted for YFP and a human cell surface marker (hCD45, hCD7, or hCD19, depending on the sample). Following the sort, the cells were centrifuged at 300g, resuspended in PBS and reinjected into NSG mice.

The bone marrow was harvested from the third passage for immediate reinjection into NSG mice for the efficacy experiments (fourth passage) and MTT assay (see below).

### Mice

Unconditioned mice were seven to nine weeks old at the time of leukemia injection. Mice were observed daily and sacrificed when leukemia cells reached 50% in the peripheral blood, luminescence was nearing saturation, or they displayed clinical symptoms (hind limb paralysis, ruffled fur, respiratory distress, poor mobility), depending on the model. This study was carried out in strict accordance with the recommendations in the Guide for the Care and Use of Laboratory Animals of the National Institutes of Health. Mice were housed in an American Association of Laboratory Animal Care—accredited facility and were treated using a protocol approved by the St. Jude Animal Care and Use Committee (Protocol Number: 465–100202) in accordance with National Institutes of Health guidelines. Mice were purchased from The Jackson Laboratory: C57Bl/6J (000664), 129X1SvJ (000691), NSG (005557) and maintained under specific pathogen-free conditions. Animals were sacrificed by carbon dioxide asphyxiation, using a gradual displacement method, consistent with the American Veterinary Medical Association Guidelines for the Euthanasia of Animals: 2013 Edition. All efforts were made to minimize suffering.

### Dexamethasone & Corticosterone LC-MS

During dexamethasone treatment, blood was drawn twice weekly, just prior to the change of medicated water for measurement of dexamethasone and corticosterone. Dexamethasone and corticosterone plasma concentrations were measured using liquid chromatography with tandem mass spectrometric detection (see supplement for details).

### *Ex vivo* MTT assay

Leukemia cells from bone marrow aspirates were tested for drug sensitivity at diagnosis of ALL, as described [22] (details in Supplement). Xenograft bone marrow samples were treated similarly, although some were incubated fewer than 96 hours. If an LC50 value could not be estimated due to resistance, it was set as twice the highest concentration tested for the purpose of statistical analysis. All xenograft bone marrow samples were depleted of mCD45+ cells with magnetic separation prior to plating, according to manufacturer’s protocol (Miltenyi autoMACS and mCD45 microbeads, #130-052-301 Miltenyi Biotec GmbH, Bergisch Gladbach, Germany). We selected seven samples that were sensitive to nanomolar concentrations and one dexamethasone resistant sample which had an LC50 >15 uM to test the efficacy of continuous and discontinuous dexamethasone dosing *in vivo*.

### *In vivo* treatment

Treatment was started 3–28 days after tail vein injection of leukemia, depending on the speed of engraftment and progression of leukemia in the absence of treatment (Table 1). Mice were randomized to a treatment group based on the ventral luminescence, so all treatment group medians for luminescence were similar at start of therapy (p>0.1 by t test). Two dexamethasone treatment regimens were tested for antileukemic efficacy: continuous dexamethasone given at 4 mg/L in the drinking water, or discontinuous dexamethasone given at 8 mg/L in the drinking water for 3.5 days, followed by no dexamethasone for 3.5 days. Thus, total weekly dose of dexamethasone was equivalent between the two treatment groups. Sulfamethoxazole (600 mg/L) and trimethoprim (120mg/L; from Hi-Tech Pharmacal, Amityville, NY) was added to the drinking water for half the week; tetracycline (1 g/L, Sigma-Aldrich, St. Louis, MO) was always present. Dexamethasone sodium phosphate solution was obtained from American Pharmaceutical Partners, Inc. (Schaumburg, IL). Water bottles were changed twice weekly for all treatment groups. Assuming 5 mL intake per day [17], the discontinuously treated mice would receive 1.6–2 mg/kg/day, which is equivalent to 4.8 to 6 mg/m^2^/day in a child based on a body surface area conversion.[23] The continuously treated mice would receive 0.8–1 mg/kg/day, equivalent to 2.4 to 3 mg/m^2^/day in a child. Mice were sacrificed when they became moribund for any reason. Treatment time varied by patient sample (Table 1). Dexamethasone treatment in all animals on an experiment was stopped when toxicity reached an unacceptable level (SJE2A007) or the bioluminescent signal decreased to baseline levels in both dexamethasone treatment groups (SJTALL030, SJBALL215, SJMLL005, SJMLL009, see S1 Fig for example). Treatment was always ended at the end of a full week of treatment, so that the same total dose was administered in the two regimens for each xenograft.

**Table 1:** Median leukemia-free survival (LFS) and growth delay factor (GDF) for each murine model.

				No dex			Cont dex				Disc dex						
Leukemia sample	Dex days	LC50-D (nM)	LC50-X (nM)	N	LFS	Range	N	LFS	Range	GDF	N	LFS	Range	GDF	Cont vs Disc dex log-rank p-value	Cont vs No dex log-rank p-value	Disc vs No dex log-rank p-value
C57Bl/6J *Arf*-/- *BCR-ABL*+	3–66	NA	9.2	10	16	16–28	20	44	23–44	28	20	46	25–66	30	1	0.005	0.008
129X1SvJ *Arf*-/- *BCR-ABL*+	3–58	NA	11.1	10	16	16–21	20	30	21–42	14	20	30	23–36	14	0.5	0.0008	0.0009
SJTALL021916	14–56	0.4	23	11	27	14–32	21	37	20–56	10	22	31	21–50	4	0.2	0.002	0.011
SJTALL033	7–50	2.9	330	5	22	21–25	10	38	35–45	16	10	37	33–49	15	0.9	0.019	0.021
SJTALL030	14–35	5.1	3.1	5	59	51–86	10	95	71–109	36	10	80	64–122	21	0.7	0.031	0.064
SJMLL005	21–56	4.9	27	10	27	16–34	20	153	38–160	126	20	156	71–157	129	0.14	0.00003	0.0001
SJBALL215	28–98	0.7	50	5	53	38–65	10	NA	134	*	10	144	128–194	91	0.15	0.00065	0.016
SJE2A007	21–91	4.5	32	4	49	46–51	10	111	110–114	62	10	77	63–112	28	0.002	0.001	0.033
SJMLL009	21–42	35.3	3.2	5	27	26–27	10	NA	93–108	*	10	106	88–109	79	0.22	0.0033	0.011
SJHYPO123	21–49	>15,000	>15,000	9	42	40–44	8	46	43–53	4	8	43	40–47	1	0.066	0.004	0.21

Cont dex: mice treated continuously with 4 mg/L dexamethasone. Disc dex: mice treated discontinuously with 8 mg/L dexamethasone for half of each week. N, number of mice per treatment group. LFS, median leukemia-free survival. LFS, Range, and GDF are shown in days. Dex days indicates the days the mice were treated with dexamethasone, with the injection of leukemia being day 0 (the same total dose was administered in the two regimens for each xenograft). LC50-D indicates the dexamethasone LC50 in the patient bone marrow at the time of ALL diagnosis. LC50-X indicates the dexamethasone LC50 in the xenograft bone marrow from the passage prior to the efficacy experiment or in the case of the mouse cell lines, the passage that was injected into the mice. Details on the calculation of LFS and GDF are found in the methods. NA indicates that the median LFS could not be calculated because of too few leukemic events.

* indicates that the GDF was not calculated because the median LFS was not calculated.

### Bioluminescent imaging

*In vivo* imaging was performed with the Xenogen IVIS-200 3–5 minutes post IP injection of 100 mg/kg D-Luciferin (Caliper Life Sciences, Hopkinton, MA). The mice were anesthetized by isoflurane (2–3% in oxygen) prior to imaging. Images were acquired with small binning, beginning at one minute exposure, or less if images were saturated. Images were analyzed with Living Image 4.0 software (Caliper Life Sciences, Hopkinton, MA). Total flux measurements (photons/second) were quantified over the whole animal.

### Flow cytometry

Blood was collected from the retro-orbital sinus, facial vein, or tail vein of anesthetized mice to assess engraftment of human cells. Blood was lysed with the BD FACS Lyse Wash Assistant (BD Biosciences, San Jose, CA). Cells were stained with antibodies to human CD45 (BD Biosciences, # 561864, APC), mouse CD45 (BD Biosciences, # 559864, PE). Samples were assayed on the BD LSR II or LSR Fortessa (BD Biosciences) and data were analyzed with FlowJo version 9 (FlowJo, LLC, Ashland, OR). Samples were sorted for hCD45 and YFP on the BD Aria (BD Biosciences).

### Leukemia-free survival

Mice receiving xenografts generally did not develop overt symptoms of leukemia prior to sacrifice, so the endpoint for leukemia-free survival was based on leukemic burden determined by either flow cytometry or luminescence. For samples that were not reliably detected in the peripheral blood, a luminescence threshold was used as the endpoint. For samples reliably detectable in the blood, an hCD45+ threshold was used as the endpoint (either 25% or 50% of cells in the live lymphocyte gate). The leukemia endpoint was hCD45>50% in the live lymphocyte gate for SJTALL021916, SJTALL033, SJTALL030, SJE2A007 and hCD45>25% for SJHYPO123. The leukemia endpoint for SJMLL009 and SJBALL215 was ventral luminescence >10^10^ p/s, and for SJMLL005, it was 5 x 10^8^ p/s, based on the peak signal in the no dexamethasone group. Mice receiving the BCR-ABL+ *Arf*-/- ALL developed hind limb paralysis, and the onset of this or other overt symptoms was used as the endpoint. Mice were censored from the analysis if they were moribund for other reasons, such as infection or accidental death.

### Statistical Analysis

Statistical analysis was performed using Statistica 11 (Statsoft, Inc, Tulsa, OK), R version 3.1.1 (www.r-project.org) or GraphPad Prism version 5.02. Leukemia-free survival was compared using the log-rank test. The growth delay factor (GDF) was estimated by subtracting the median survival of the untreated group from that of each treated group. Spleen weights and LC50 values were compared with the Mann Whitney test. P values less than 0.05 were considered statistically significant and were not adjusted for multiple comparisons.

## Results

**Fig 1:**
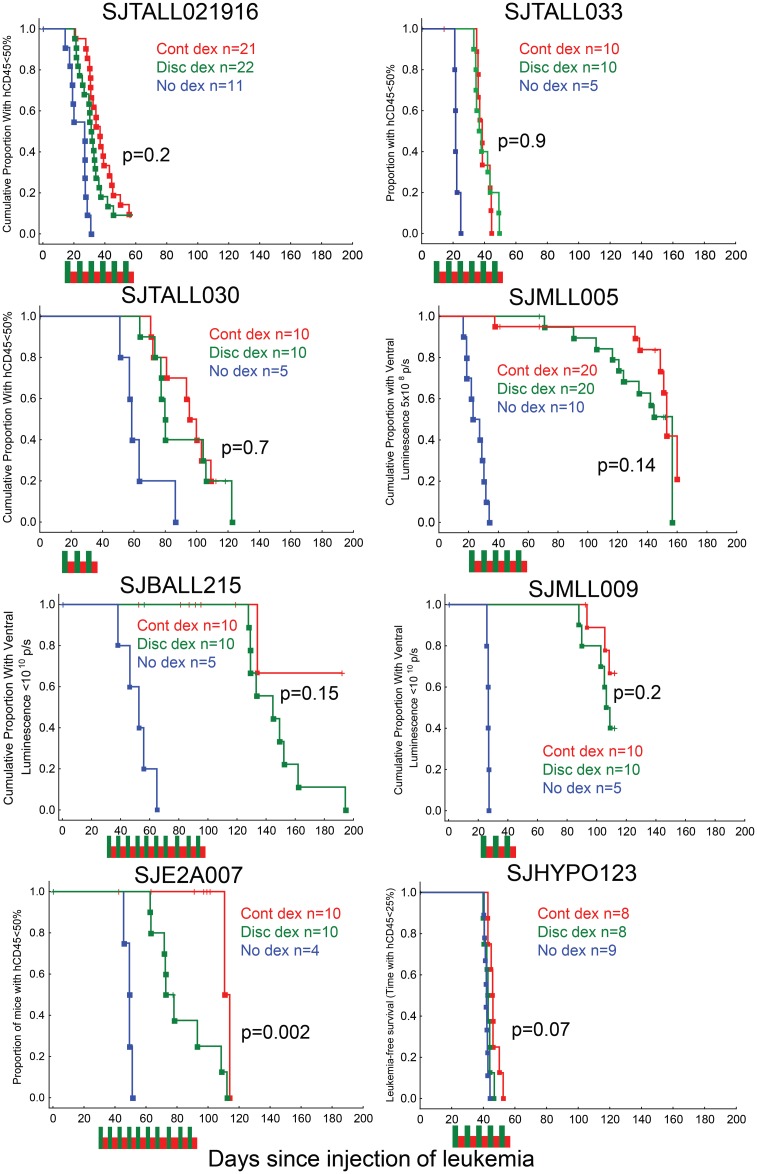
Antileukemic efficacy of continuous, discontinuous, and no dexamethasone for xenografted patient ALL samples. Red indicates continuous dexamethasone (Cont dex), green indicates discontinuous dexamethasone (disc dex), and blue indicates no dexamethasone. Squares on the curve indicate leukemic events, and + indicates censoring due to toxicity, accidental death, or the end of the experiment. Red and green boxes indicate treatment period—red indicates continuous dexamethasone (4 mg/L) and green indicates discontinuous dexamethasone (8 mg/L for 3.5 days, 0 for 3.5 days). P-values shown were calculated with the log-rank test, comparing the continuous and discontinuous dexamethasone treatment arms, see Table 1 for comparisons of dexamethasone vs no dexamethasone.

For all dexamethasone-sensitive xenograft samples, there was improved leukemia-free survival (LFS) with at least one of the dexamethasone treatment regimens compared to no dexamethasone treatment (median LFS +4 to +129 days relative to no dexamethasone, log-rank p = 0.064 to p = 0.00003, Fig 1, Table 1). The dexamethasone-resistant sample did not have improved LFS with discontinuous dexamethasone (p = 0.21, +1 day), but did with continuous dexamethasone (p = 0.004, +4 days), although this small improvement in survival is likely biologically insignificant. Four of the samples reached the leukemic endpoint during dexamethasone treatment (SJTALL021916, SJTALL033, SJE2A007, and SJHYPO123), while the others reached this endpoint only after dexamethasone was stopped (e.g. SJMLL009, S1 Fig).

**Fig 2:**
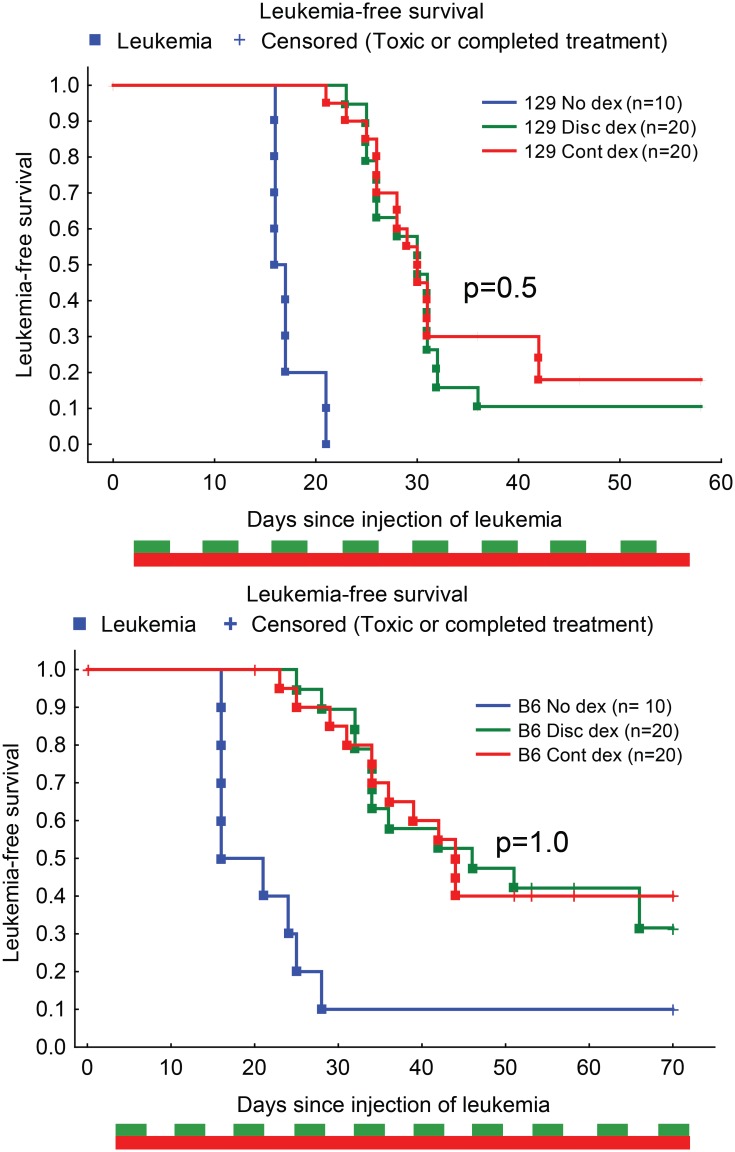
Anti-leukemic survival was equivalent between dexamethasone treatment regimens in the murine ALL model. Mice of the 129X1SvJ strain (A) and C57Bl/6 strain (B) were injected with *Arf*-/- BCR-ABL+ luciferase+ leukemia and treated with continuous (Cont dex, red), discontinuous dexamethasone (Disc dex, green), or no dexamethasone (blue) regimens. Boxes indicate leukemic events, while + indicates censored events. P-values indicated are log-rank p-values comparing the continuous and discontinuous dexamethasone regimens, see Table 1 for comparisons of dexamethasone vs no dexamethasone.

The murine C57Bl/6J and 129X1SvJ *Arf*-/- BCR-ABL preB cell lines were sensitive to dexamethasone, with LC50s of 9.2 (±0.6), and 11.1 (±0.6) nM, respectively (S2 Fig). LFS was improved with dexamethasone compared to no dexamethasone (median LFS +30 to +46 days, p<0.01 for all comparisons). There was equivalent efficacy of the two dexamethasone regimens in both murine BCR-ABL strains (p = 0.5 and p = 1.0, respectively), which were significant improvements over no dexamethasone treatment (Fig 2, Table 1). Both strains reached the leukemic endpoint during dexamethasone treatment. The growth delay factors (GDF) were similar for the continuous and discontinuous regimens in the murine BCR-ABL model for both the C57Bl/6J and 129X1SvJ strains, but differed between strains (28–30 days for C57Bl/6J and 14 days for 129X1SvJ, Table 1).

**Fig 3:**
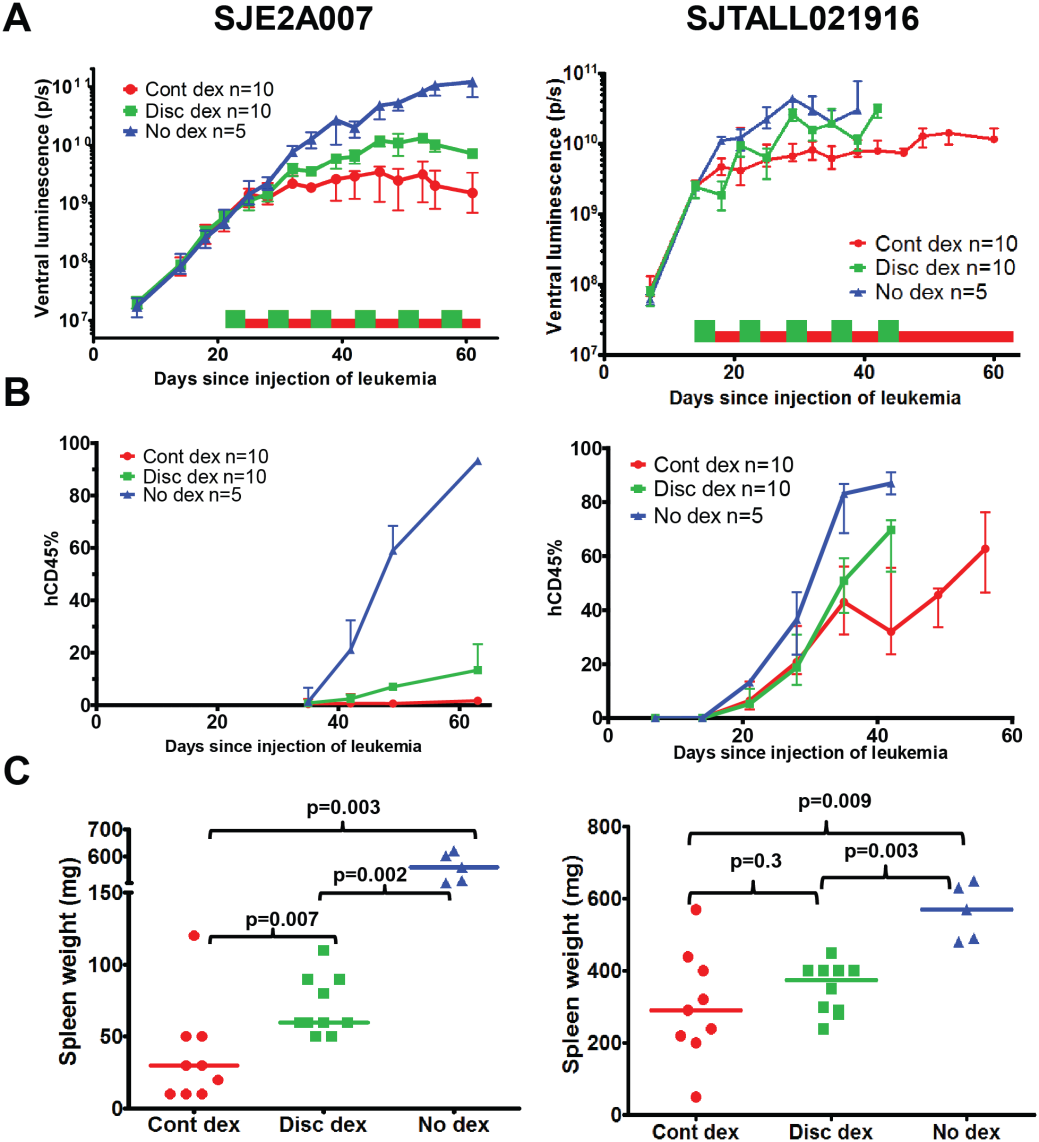
Continuous dexamethasone was more effective than discontinuous dexamethasone for SJE2A007 cells but not SJTALL021916 cells. Red indicates continuous dexamethasone (Cont dex), green indicates discontinuous dexamethasone (disc dex), and blue indicates no dexamethasone. A, ventral luminescence over time. Symbols indicate median luminescence and error bars indicate interquartile range. Red and green boxes near the x-axis indicate treatment period (continuous dexamethasone in red, discontinuous dexamethasone in green). B, hCD45% in the blood over time. Symbols indicate median and error bars indicate interquartile range. C, spleen weight on day 62 (SJE2A007, left) or at the time of sacrifice (SJTALL021916, right). Symbols indicate individual mice, and horizontal line indicates median. P values were calculated with a two-tailed Mann Whitney test.

For seven of the eight xenograft samples, including the dexamethasone resistant sample, there were no significant differences in LFS between the continuous and discontinuous regimens (median LFS differed by 1–15 days, log-rank p = 0.066 to 0.9, Table 1 & Fig 1). For one of the eight samples (SJE2A007), the continuous dexamethasone was more effective than the discontinuous schedule (median LFS of 111 vs 77 days, log-rank p = 0.002). In a second experiment with the same sample, all the mice were sacrificed on the same day from start of therapy. ALL burden was lower in the continuous dexamethasone group than the discontinuous dexamethasone group as assessed by ventral luminescence (Kruskal-Wallis p = 0.004, Fig 3A, S3 Fig), hCD45% in the peripheral blood (Kruskal-Wallis p = 0.007, Fig 3B) and by spleen size (Kruskal-Wallis p = 0.006, Fig 3C). For two of the three T cell ALL samples (SJTALL021916 and SJTALL033), there was a cyclical pattern in the luminescence of the discontinuously treated group, in which it increased during the steroid holiday and decreased or remained constant during the time the dexamethasone was in the drinking water (Fig 3A).

**Fig 4:**
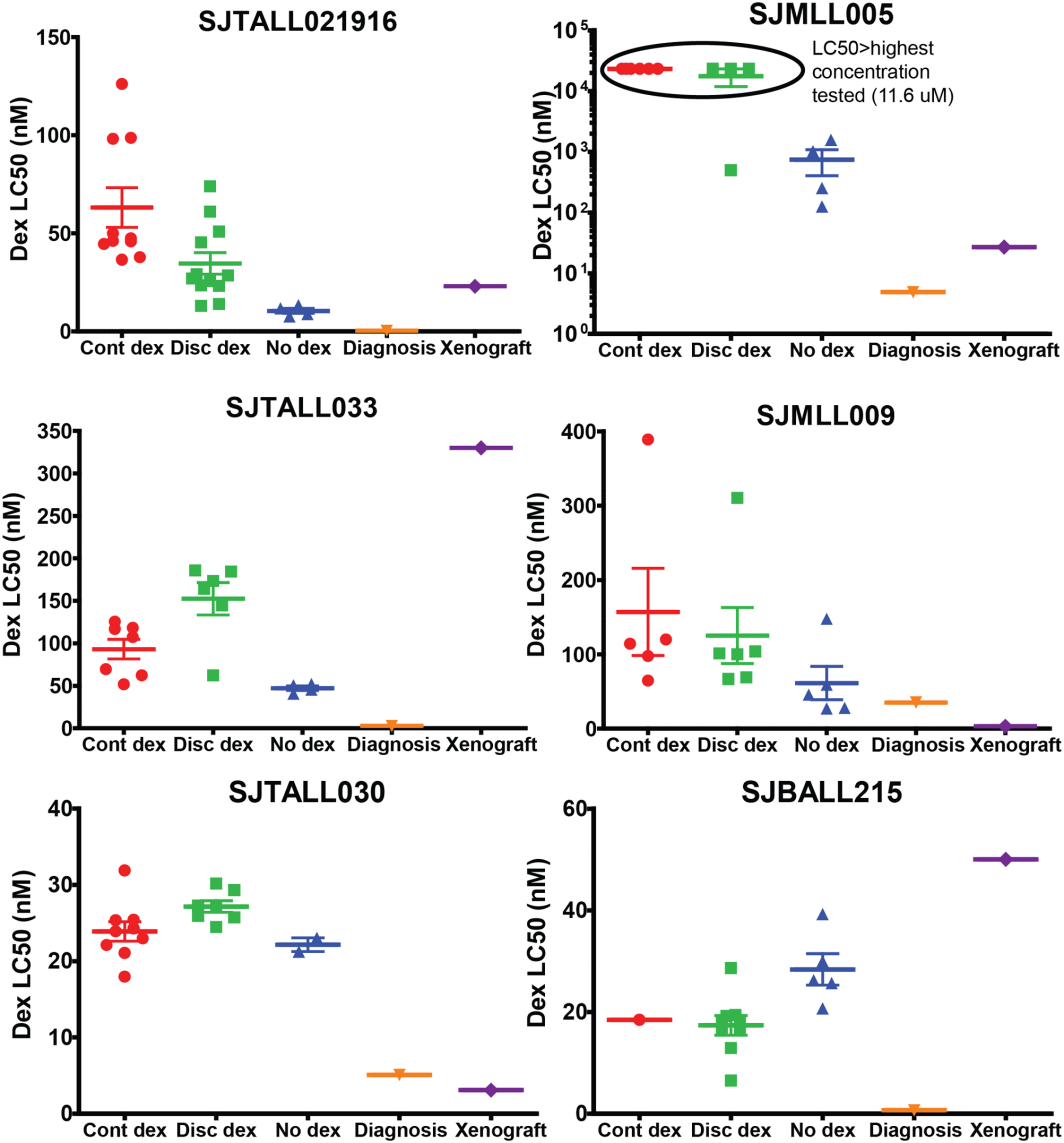
Leukemia cells generally became less sensitive to dexamethasone after *in vivo* treatment. Dexamethasone sensitivity of leukemic bone marrow cells at diagnosis (orange), the passage prior to *in vivo* treatment (purple), or at the time of sacrifice after *in vivo* treatment (continuous dexamethasone, red; discontinuous dexamethasone, green; no dexamethasone, blue). Symbols indicate individual mice, the horizontal line indicates the mean, and error bars indicate standard error of the mean (SEM). Incubation time for all diagnosis samples in the MTT assay was 4 days. Incubation time for cells from the mice receiving continuous dexamethasone, discontinuous dexamethasone, and no dexamethasone was 4 days for SJTALL021916, SJTALL033, SJBALL215, 3 days for SJTALL030, and 2 days for SJMLL005 and SJMLL009. *Ex vivo* sensitivity was not tested in SJE2A007 due to poor viability after two days *ex vivo* (<50%), and SJHYPO123 due to resistance of the sample at the time of diagnosis (LC50 > highest concentration tested, 15 uM, which was recapitulated in the xenograft sample prior to *in vivo* treatment).

The *ex vivo* sensitivity was tested after *in vivo* dexamethasone treatment for six of the eight xenograft samples (Fig 4). The remaining two samples were not tested because of poor viability *ex vivo* (SJE2A007, <50% viable at 2 days *ex vivo*) or lack of sensitivity *ex vivo* (SJHYPO123, LC50 >15uM). The *ex vivo* sensitivity to four days of dexamethasone of the xenograft samples pre-treatment or with no dexamethasone treatment was similar to that measured in the original diagnosis sample in the majority of cases tested, however, this comparison is complicated by the fact that the duration of the exposure to dexamethasone had to be reduced in some of the xenograft samples that didn’t survive well *in vitro* for four days. The SJMLL005 sample may have been more resistant after multiple passages *in vivo*, but it was tested for only two days *in vitro* versus four days for the diagnosis sample. The SJTALL021916 cells were more resistant *ex vivo* after *in vivo* dexamethasone treatment (within the same passage), and the leukemic cells recovered from continuously treated mice had higher LC50s than those recovered from the discontinuously treated animals (63.2 nM vs 34.6 nM, p = 0.02), which were both higher than the leukemic cells from the mice receiving no dexamethasone (10.5 nM, both p<0.01). The SJTALL033 cells were more resistant *ex vivo* after *in vivo* dexamethasone treatment (both p<0.02 when compared with the mice receiving no dexamethasone in the same passage, 47.3 nM), although the discontinuously treated mice had higher LC50s than the continuously treated mice (169 vs 107 nM, p = 0.04). The SJTALL030 cells had LC50s in the 20–30 nM range, after discontinuous or continuous therapy) as well as from the mice receiving no dexamethasone. SJMLL005 cells also appeared more resistant to dexamethasone *ex vivo* after *in vivo* dexamethasone treatment, though the cells from the dexamethasone-treated mice did not reach 50% of the control viability, so an LC50 was not calculated. The SJMLL009 cells from all treatment groups had similar LC50s, although the trend was for higher values in the dexamethasone-treated animals versus no dexamethasone. The SJBALL215 cells from dexamethasone-treated mice had lower LC50s than the mice that received no dexamethasone (17 nM vs 28 nM, p = 0.007).

**Fig 5:**
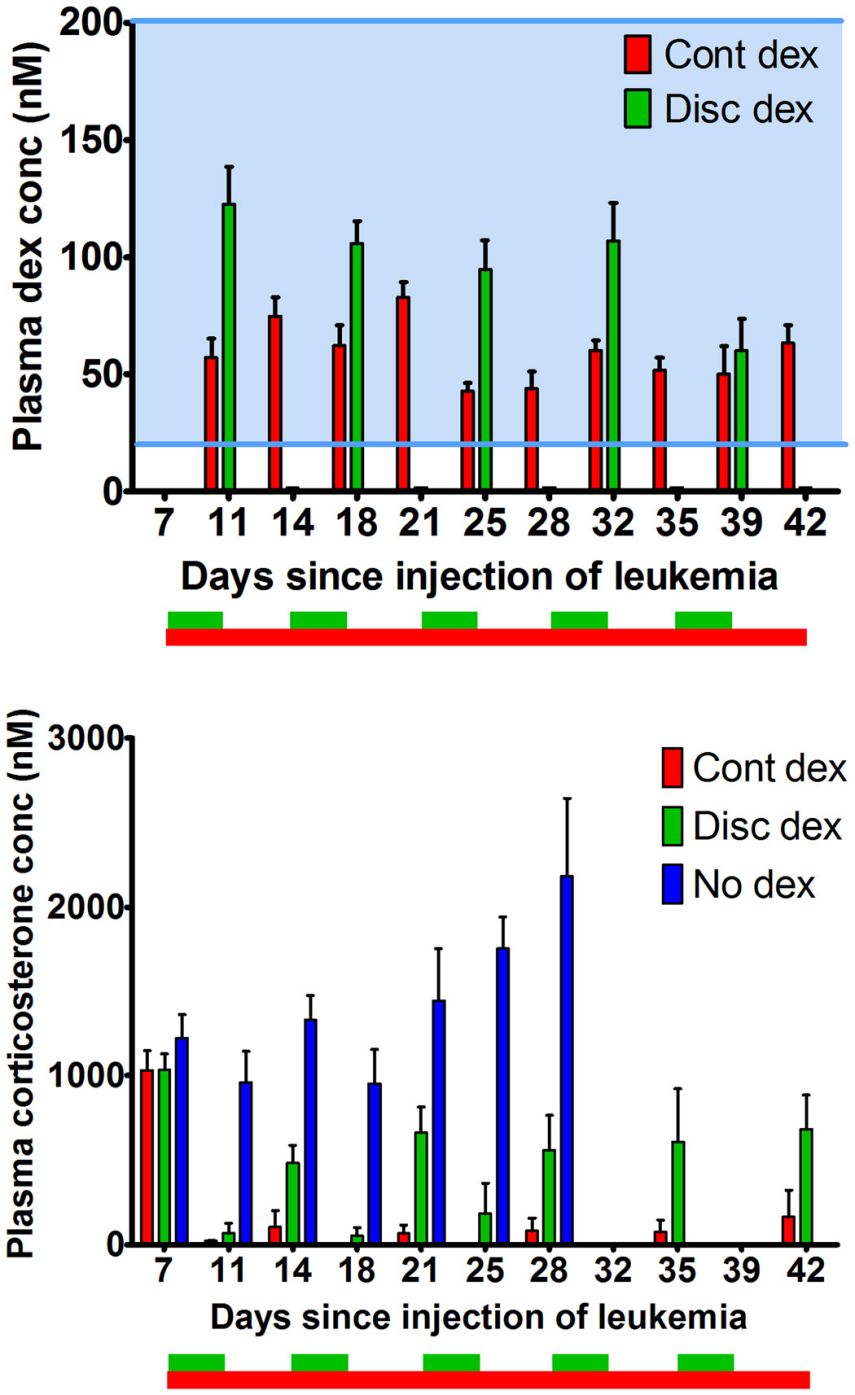
Plasma dexamethasone concentrations in mice during treatment were similar to those achieved in patients and corticosterone was suppressed in dexamethasone treated mice. Plasma dexamethasone (A) and corticosterone concentrations (B) in mice engrafted with SJTALL033 cells. Blue shaded area indicates the concentration of dexamethasone found in patient samples (20–200 nM). Plasma dexamethasone (dex) concentrations were determined by LC-MS twice weekly, just prior to the change of medicated water. N = 10 in the dexamethasone treatment groups and N = 5 in the untreated controls (no dex group). Treatment began on day 7 after plasma was taken, and is indicated by the boxes below the x-axis. Green indicates discontinuous dexamethasone and red indicates continuous dexamethasone. Dexamethasone was in the drinking water of the discontinuous (disc) dexamethasone group from day 7–10.5, 14–17.5, 21–24.5, 28–31.5, and 35–39.5. Bar height indicates mean and error bars indicate standard error of the mean (SEM).

Plasma concentrations of dexamethasone in mice on treatment were similar to plasma levels (20–200 nM) achieved in patients treated for ALL [24] (Fig 5). On days that dexamethasone was included in the drinking water of the discontinuously dosed mice, dexamethasone concentrations were higher in the discontinuously treated group than the continuously treated group (Fig 5A), which was expected since the concentration in the discontinuously treated drinking water was twice the concentration in the continuously treated group (8 mg/L vs 4 mg/L). The SJTALL033 cells proliferated *in vivo* through dexamethasone treatment (Fig 1), and the *ex vivo* sensitivity assay demonstrated LC50 levels at or above the mean plasma concentration (Figs 4 and 5). On days that the dexamethasone was not included in the drinking water, corticosterone levels in the discontinuously treated group were lower than the no dexamethasone group, indicating the corticosterone levels had not completely recovered in 3.5 days off therapy (Fig 5B). Dexamethasone concentrations were not detectable in the mice on the discontinuous regimen after 3.5 days off therapy. The mice receiving continuous dexamethasone had plasma dexamethasone concentrations of 40–90 nM, and undetectable levels of corticosterone at all time points after dexamethasone treatment began, whereas mice in the discontinuous regimen had some recovery of corticosterone during dexamethasone holidays.

## Discussion

Because the cumulative incidence of symptomatic osteonecrosis in ALL patients can be as high as 10–20%,[1–7] efforts should be made to minimize treatment-related risk factors that may induce osteonecrosis without compromising antileukemic efficacy. Previously, we’ve shown that the incidence of osteonecrosis is less in mice receiving dexamethasone discontinuously versus continuously,[17] supporting the clinical findings that discontinuous dexamethasone treatment is associated with lower risk of osteonecrosis. However, the impact of discontinuous dexamethasone on antileukemic efficacy has heretofore not been evaluated. Others have previously shown the clinical relevance of ALL xenograft models by demonstrating that the time to engraftment is correlated with relapse risk,[25] the global gene expression of the xenografted leukemia is similar to the primary sample [26] and the xenograft’s *in vivo* response to dexamethasone correlated with patient outcome.[27]

Herein, we have demonstrated that there is no difference in the antileukemic efficacy of continuous and discontinuous dexamethasone regimens in the majority of murine and human ALL models. All patients whose samples were used in this study remain in remission, including the single patient whose ALL did show superior efficacy with continuous dexamethasone (SJE2A007). These data indicate that for most pediatric ALL, discontinuous dexamethasone is likely to be as effective as continuous dexamethasone; however, it is possible that for some ALL cases, there may be inferior efficacy with discontinuous dexamethasone.

We have shown that the doses the mice received yielded comparable plasma dexamethasone concentrations to those achieved in patients with ALL.[17] Dosing the dexamethasone in the drinking water provides a level of systemic exposure that is similar to what the patients receive; because of dexamethasone’s relatively long half-life, with doses split into two or three oral doses per day, patients maintain near-constant plasma levels of drug during dexamethasone pulses.[17, 28] Synthetic glucocorticoids such as dexamethasone suppress the release of naturally occurring glucocorticoids: cortisol in humans, and corticosterone in mice. The mice receiving continuous dexamethasone had nearly complete corticosterone suppression throughout therapy, while the mice receiving discontinuous dexamethasone start producing corticosterone while they are not receiving dexamethasone, although it does not reach the concentration seen prior to treatment or in the untreated group of mice. Because cortisol suppression by glucocorticoids in humans has been related to adverse effects such as growth suppression,[29, 30] this may be a useful biomarker in studies of schedule-dependent glucocorticoid toxicity.

We have shown previously that age, gender, strain, and substrain (vendor) influence the incidence of dexamethasone-induced osteonecrosis in mice, with the highest incidence in male BALB/cJ mice started on treatment at 4 weeks of age.[17, 31] However, mice this young are not optimal for evaluation of antileukemic efficacy. In our current study, because the mice were older (8–12 weeks) when treatment started, and most had leukemia complicating evaluation of bone histology at the time of sacrifice, they were not evaluable for osteonecrosis. The doses used in this study (4 mg/L continuously vs 8 mg/L discontinuously) are exactly twice the doses used in the young BALB/cJ mice for definitive osteonecrosis studies, which demonstrate that continuous exposure is more osteonecrotic than discontinuous exposure; although these doses produce osteonecrosis, they also cause morbidity and mortality from opportunistic infections which precludes unbiased evaluations of osteonecrosis. The corticosterone recovery after 3.5 days off treatment was observed in both the NSG mice with leukemia treated with higher doses and the young BALB/cJ mice treated with lower doses.[17]

Most of the xenograft samples were more resistant to dexamethasone *ex vivo* after *in vivo* dexamethasone treatment, although this wasn’t the case for every sample, and some exhibited minimal changes in LC50. We acknowledge that we may have selected different clones in the xenograft samples than were present in the diagnosis sample through passaging in mice, lentiviral transduction, sorting, and dexamethasone treatment. Other investigators have used xenografts to demonstrate that *ex vivo* dexamethasone sensitivity and *in vivo* LFS correlate well,[32] but clonal selection can occur in xenografts and influence dexamethasone sensitivity.[33, 34] It is also possible the leukemia was epigenetically reprogrammed, which is one mechanism of glucocorticoid resistance.[35–37] Nonetheless, most samples were equally sensitive to continuous vs discontinuous dexamethasone post *in vivo* treatment. The start of treatment and duration varied by sample due to the variable time to engraftment and response to treatment (Table 1). This might theoretically impact outcome by selecting more resistant clones with longer treatment duration, however, we did not observe such an effect in our study.

For two T-ALL samples (SJTALL021916 and SJTALL033), there was a cyclical pattern in the ventral luminescence, indicating that during the 3.5 days when the dexamethasone was stopped, the cells grew very quickly, and when the mice were receiving dexamethasone, the cells grew more slowly. When tested for *ex vivo* dexamethasone LC50, one sample (SJTALL033) exhibited a higher LC50 after discontinuous than continuous treatment, and the opposite was seen for the SJTALL021916 cells. Thus, although discontinuous dexamethasone may permit cells time to recover between pulses, this did not necessarily result in more dexamethasone resistance.

Although discontinuous glucocorticoids have been used in many ALL regimens and for many other diseases in an attempt to reduce adverse effects [38–46], there are almost no data that compare disease efficacy in continuous versus discontinuous regimens. There has been one clinical trial that randomized pediatric ALL patients to receive continuous vs discontinuous dexamethasone during reinduction phases of therapy (CCG-1961), but the survival data have been incompletely evaluated. Although the study suggests that there was no difference in outcome between continuous and discontinuous dexamethasone in the context of an additional delayed intensification phase,[8, 15, 16] there has also been a suggestion that patients who developed osteonecrosis had higher LFS by some [15] but not others.[3] Discontinuous dexamethasone in post-induction therapy is now commonly used in clinical trials;[47–50] however, it is not universally accepted,[51, 52] and daily glucocorticoids for four weeks remains standard for most ALL induction regimens. Thus, it is highly relevant to compare the efficacy of continuous versus discontinuous dexamethasone in and experimental model of ALL in which the dexamethasone schedule can be systematically studied. Our results suggest there will be equal efficacy of continuous and discontinuous dexamethasone for the majority of patients with ALL.

## Supporting Information

S1 Fig Ventral luminescence in mice injected with SJMLL009 cells decreased during treatment and increased when treatment was discontinued. Treatment was started at day 21 and ended day 42 after injection of leukemia because the luminescence signal had decreased to baseline levels. Treatment periods are indicated by the red and green boxes below the x-axis. Green indicates discontinuous dexamethasone, red indicates continuous dexamethasone, and blue indicates no dexamethasone. Each line represents one mouse.
(DOCX)

S2 Fig *In vitro* sensitivity of murine BCR-ABL cell lines to dexamethasone. Blue indicates BCR-ABL+ Arf-/- cells of the 129X1SvJ background, and red indicates BCR-ABL+ Arf-/- cells on the C57BL/6 background. Sensitivity was tested with the MTS assay and LC50 was calculated using a four parameter logistic model (details in S1 Methods).
(DOCX)

S3 Fig Luminescence at day 61 of mice injected with SJE2A007 cells shows that continuous dexamethasone is more effective than discontinuous dexamethasone. Mice treated with no dexamethasone are in the top row, mice in the discontinuous dexamethasone treatment group are in the second and third rows, and mice in the continuous dexamethasone treatment group are in the fourth and fifth rows. Images were acquired at 1 second exposure. The color scale was set to 5x10^7 –^ 2x10^9^, and smoothing was set to 3x3 for each image.
(DOCX)

S1 Methods Detailed methods of LC-MS assay of dexamethasone and corticosterone, *ex vivo* MTT assay, and *in vitro* MTS assay. (DOCX)

S1 Table Patient demographics. All patients were treated on either the St. Jude Total XV or XVI protocols and remain in remission. Risk Group classification has been described previously for Total XV [48] and XVI [49] protocols.
(DOCX)
