# Supplementary material for: Antileukemic Efficacy of Continuous vs Discontinuous Dexamethasone in Murine Models of Acute Lymphoblastic Leukemia
Source: PLoS One. 2015 Aug 7;10(8):e0135134. doi: 10.1371/journal.pone.0135134 (PMC4529108; doi:10.1371/journal.pone.0135134)
Supplement: S1 Fig — Treatment was started at day 21 and ended day 42 after injection of leukemia because the luminescence signal had decreased to baseline levels. Treatment periods are indicated by the red and green boxes below the x-axis. Green indicates discontinuous dexamethasone, red indicates continuous dexamethasone, and blue indicates no dexamethasone. Each line represents one mouse. (DOCX) [file pone.0135134.s001.docx]

Supplement to Antileukemic efficacy of continuous vs discontinuous dexamethasone in murine models of acute lymphoblastic leukemia

Laura B. Ramsey^1^, Laura J. Janke^2^, Monique A. Payton^1^, Xiangjun Cai^1^, Steven W. Paugh^1^, Seth E. Karol^1^, Landry Kamdem Kamdem^3^, Cheng Cheng^4^, Richard T. Williams^5^, Sima Jeha^6^, Ching-Hon Pui^6^, William E. Evans^1^, Mary V. Relling^1*^

^1^Pharmaceutical Sciences Department, St. Jude Children’s Research Hospital, Memphis, TN, USA;

^2^Department of Pathology, St. Jude Children’s Research Hospital, Memphis, TN, USA;

^3^Harding University College of Pharmacy, Searcy, AR, USA;

^4^Biostatistics Department, St. Jude Children’s Research Hospital, Memphis, TN, USA;

^5^Puma Biotechnology Inc., Los Angeles, CA, USA;

^6^Department of Oncology, St. Jude Children’s Research Hospital, Memphis, TN, USA.

* Corresponding author:

Email: mary.relling@stjude.org (MVR)

**S1 Fig. Ventral luminescence in mice injected with SJMLL009 cells decreased during treatment and increased when treatment was discontinued.** Treatment was started at day 21 and ended day 42 after injection of leukemia because the luminescence signal had decreased to baseline levels. Treatment periods are indicated by the red and green boxes below the x-axis. Green indicates discontinuous dexamethasone, red indicates continuous dexamethasone, and blue indicates no dexamethasone. Each line represents one mouse.
