# Supplementary material for: Antileukemic Efficacy of Continuous vs Discontinuous Dexamethasone in Murine Models of Acute Lymphoblastic Leukemia
Source: PLoS One. 2015 Aug 7;10(8):e0135134. doi: 10.1371/journal.pone.0135134 (PMC4529108; doi:10.1371/journal.pone.0135134)
Supplement: S2 Fig — Blue indicates BCR-ABL+ Arf-/- cells of the 129X1SvJ background, and red indicates BCR-ABL+ Arf-/- cells on the C57BL/6 background. Sensitivity was tested with the MTS assay and LC50 was calculated using a four parameter logistic model (details in S1 Methods). (DOCX) [file pone.0135134.s002.docx]

Supplement to Antileukemic efficacy of continuous vs discontinuous dexamethasone in murine models of acute lymphoblastic leukemia

Laura B. Ramsey^1^, Laura J. Janke^2^, Monique A. Payton^1^, Xiangjun Cai^1^, Steven W. Paugh^1^, Seth E. Karol^1^, Landry Kamdem Kamdem^3^, Cheng Cheng^4^, Richard T. Williams^5^, Sima Jeha^6^, Ching-Hon Pui^6^, William E. Evans^1^, Mary V. Relling^1*^

^1^Pharmaceutical Sciences Department, St. Jude Children’s Research Hospital, Memphis, TN, USA;

^2^Department of Pathology, St. Jude Children’s Research Hospital, Memphis, TN, USA;

^3^Harding University College of Pharmacy, Searcy, AR, USA;

^4^Biostatistics Department, St. Jude Children’s Research Hospital, Memphis, TN, USA;

^5^Puma Biotechnology Inc., Los Angeles, CA, USA;

^6^Department of Oncology, St. Jude Children’s Research Hospital, Memphis, TN, USA.

* Corresponding author:

Email: mary.relling@stjude.org (MVR)

**S2 Fig.** ***In vitro* sensitivity of murine BCR-ABL cell lines to dexamethasone.** Blue indicates BCR-ABL+ Arf-/- cells of the 129X1SvJ background, and red indicates BCR-ABL+ Arf-/- cells on the C57BL/6 background. Sensitivity was tested with the MTS assay and LC50 was calculated using a four parameter logistic model (details in S1 Methods).
