# Supplementary material for: Antileukemic Efficacy of Continuous vs Discontinuous Dexamethasone in Murine Models of Acute Lymphoblastic Leukemia
Source: PLoS One. 2015 Aug 7;10(8):e0135134. doi: 10.1371/journal.pone.0135134 (PMC4529108; doi:10.1371/journal.pone.0135134)
Supplement: S3 Fig — Mice treated with no dexamethasone are in the top row, mice in the discontinuous dexamethasone treatment group are in the second and third rows, and mice in the continuous dexamethasone treatment group are in the fourth and fifth rows. Images were acquired at 1 second exposure. The color scale was set to 5x10^7^ – 2x10^9^, and smoothing was set to 3x3 for each image. (DOCX) [file pone.0135134.s003.docx]

Supplement to Antileukemic efficacy of continuous vs discontinuous dexamethasone in murine models of acute lymphoblastic leukemia

Laura B. Ramsey^1^, Laura J. Janke^2^, Monique A. Payton^1^, Xiangjun Cai^1^, Steven W. Paugh^1^, Seth E. Karol^1^, Landry Kamdem Kamdem^3^, Cheng Cheng^4^, Richard T. Williams^5^, Sima Jeha^6^, Ching-Hon Pui^6^, William E. Evans^1^, Mary V. Relling^1*^

^1^Pharmaceutical Sciences Department, St. Jude Children’s Research Hospital, Memphis, TN, USA;

^2^Department of Pathology, St. Jude Children’s Research Hospital, Memphis, TN, USA;

^3^Harding University College of Pharmacy, Searcy, AR, USA;

^4^Biostatistics Department, St. Jude Children’s Research Hospital, Memphis, TN, USA;

^5^Puma Biotechnology Inc., Los Angeles, CA, USA;

^6^Department of Oncology, St. Jude Children’s Research Hospital, Memphis, TN, USA.

* Corresponding author:

Email: mary.relling@stjude.org (MVR)


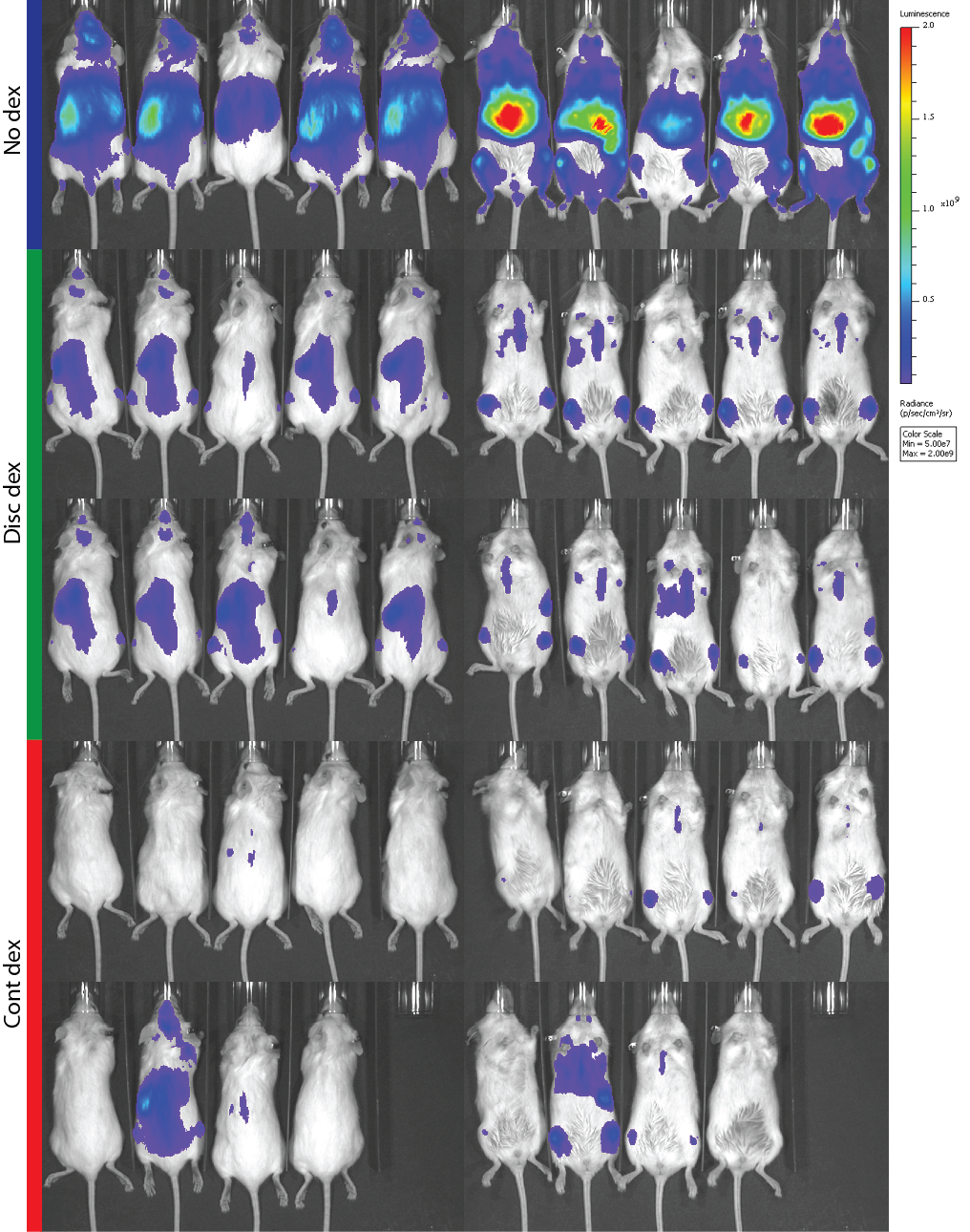


**S3 Fig. Luminescence at day 61 of mice injected with SJE2A007 cells shows that continuous dexamethasone is more effective than discontinuous dexamethasone.** Mice treated with no dexamethasone are in the top row, mice in the discontinuous dexamethasone treatment group are in the second and third rows, and mice in the continuous dexamethasone treatment group are in the fourth and fifth rows. Images were acquired at 1 second exposure. The color scale was set to 5x10^7^ – 2x10^9^, and smoothing was set to 3x3 for each image.
