# Supplementary material for: Antileukemic Efficacy of Continuous vs Discontinuous Dexamethasone in Murine Models of Acute Lymphoblastic Leukemia
Source: PLoS One. 2015 Aug 7;10(8):e0135134. doi: 10.1371/journal.pone.0135134 (PMC4529108; doi:10.1371/journal.pone.0135134)
Supplement: S1 Methods — (DOCX) [file pone.0135134.s004.docx]

Supplement to Antileukemic efficacy of continuous vs discontinuous dexamethasone in murine models of acute lymphoblastic leukemia

Laura B. Ramsey^1^, Laura J. Janke^2^, Monique A. Payton^1^, Xiangjun Cai^1^, Steven W. Paugh^1^, Seth E. Karol^1^, Landry Kamdem Kamdem^3^, Cheng Cheng^4^, Richard T. Williams^5^, Sima Jeha^6^, Ching-Hon Pui^6^, William E. Evans^1^, Mary V. Relling^1*^

^1^Pharmaceutical Sciences Department, St. Jude Children’s Research Hospital, Memphis, TN, USA;

^2^Department of Pathology, St. Jude Children’s Research Hospital, Memphis, TN, USA;

^3^Harding University College of Pharmacy, Searcy, AR, USA;

^4^Biostatistics Department, St. Jude Children’s Research Hospital, Memphis, TN, USA;

^5^Puma Biotechnology Inc., Los Angeles, CA, USA;

^6^Department of Oncology, St. Jude Children’s Research Hospital, Memphis, TN, USA.

* Corresponding author:

Email: mary.relling@stjude.org (MVR)

**Supplemental Methods**

**Dexamethasone & Corticosterone LC-MS**

To 10 µL of sample, 50 µL of internal standard, 50 ng/mL isotope labeled d4_Dexamethasone in acetonitrile (ACN), was added and vortexed for 15 min at 1700rpm using a DVX-2500 multi-tube vortexer from VWR (Radnor, PA). After centrifugation at 16,000g for 8 min at 4 º C, the supernatant was transferred to an autosampler vial and 3 µl of the solution was injected onto the HPLC-MS system. The chromatographic separation was achieved by using a Waters UPLC with Waters ACQUITY BEHC18 column (1.7 µm, 100 x 2.1 mm), and protected by an ACQUITY in-line filter. The column was maintained at 40 ± 5 º C and autosampler at 15 ± 5 ºC. The mobile phase was generated by mixing 10mM ammonium acetate in H_2_O with ACN. The flow rate was 0.7 mL/min. The linear gradient was started at time zero from 30% ACN and 70% buffer to 70% ACN and 30% buffer in 1.2 min, and then returned to the starting condition in a 0.01-minute step, followed by a 1.79 min hold for equilibrium. The injection interval was 3.2 min. Detection of analytes was performed with a Waters TQD mass spectrometer operated in the positive MRM mode using Masslynx 4.1 software. The following mass ions (m/z) were used for detection: m/z 393.10>373.03 for dexamethasone; m/z 347.10>120.96 for corticosterone, and m/z 397.10>377.09 for internal standard. The following tune parameters were retained for optimal detection: capillary voltage, 0.5 kV; cone voltage, 20 V for dexamethasone, 34 V for corticosterone, and 22 V for internal standard; source temperature, 150°C; desolvation temperature, 450 º C; cone gas flow, 5 L/h; desolvation gas flow, 800 L/h and collision energy, 8 eV for dexamethasone, 30 eV for corticosterone, and 10 eV for internal standard.

Standards were prepared in stripped mouse plasma over the linear ranges of 2 - 500 ng/ml (5.1 - 1275 nM) and 4 - 1000 ng/ml (11.5 - 2886 nM) for dexamethasone and corticosterone respectively. The interday and intraday imprecisions of dexamethasone at 6, 80, 400 ng/mL (15.3, 204, and 1020 nM) and corticosterone at 15, 80, 800 ng/mL (43.3, 231, and 2309 nM) were all <8.9% and the accuracy was 98.3%-114%. At the limit of quantitation, the interday and intraday imprecision was <14% and accuracy was better than 88%. No interference from endogenous compounds was observed. Dexamethasone, corticosterone, and ammonium acetate (98%, v/v in water) were purchased from Sigma Aldrich (St. Louis, MO). d4_Dexamethasone was from CDN Isotopes Inc (Pointe-Claire, Quebec, Canada). ACN was of HPLC grade and obtained from Burdick & Jackson (Muskegon, MI).

***Ex vivo* MTT assay**

Cells were plated in RPMI-1640 with 2 mM L-glutamine, 20% heat-inactivated fetal bovine serum, 1X Antibiotic-Antimycotic solution (Life Technologies, Grand Island, NY) and 1X ITS solution (Life Technologies, Grand Island, NY) at a concentration of two million cells per milliliter, with 80 uL in each experimental well of a round-bottom 96-well plate. Twenty microliters of decreasing concentrations of dexamethasone were added and the plates were incubated for 96 hours in a humidified incubator containing 5% CO2 at 37°C. For the final six hours, ten microliters of 5 mg/mL MTT (3-4,5-dimethylthiazol-2,5-diphenyl tetrazolium bromide) was added to each experimental well. Crystals were solubilized with 100 uL acidified isopropyl alcohol (0.04N HCl) and mixed well. Plates were read at 570 nm with reference at 720 nm. LC50 values were determined as previously described ^1^ using ADAPT II modeling software (Biomedical Simulations Resource, Los Angeles, CA)^2^.

***In vitro* MTS assay**

The CellTiter 96 AQueous Non-Radioactive Cell Proliferation Assay (Promega, Madison, WI) was used to assess the sensitivity of the murine Arf-/- BCR-ABL+ cell lines to dexamethasone. 10,000 cells were added to wells of a 96-well plate and incubated in media overnight. Dexamethasone sodium phosphate was added 24 hours later. After an additional 72 hours of incubation, the MTS reagent was added. Plates were read at 490 nm after 1.5 – 2 hours of incubation. A four parameter logistic model was used to fit the concentration versus viability data and estimate the LC50 (concentration lethal to 50% of the treated cells compared to untreated control cells).

**References**

1. Holleman A, Cheok MH, den Boer ML, et al: Gene-expression patterns in drug-resistant acute lymphoblastic leukemia cells and response to treatment. N Engl J Med 351:533-42, 2004

2. D’Argenio DZaAS: ADAPT II User’s Guide: Pharmacokinetic/Pharmacodynamic Systems Analysis Software., Biomedical Simulations Resource, Los Angeles, 1997

3. Pui CH, Campana D, Pei D, et al: Treating childhood acute lymphoblastic leukemia without cranial irradiation. N Engl J Med 360:2730-41, 2009

4. Jeha S, Pui CH: Risk-adapted treatment of pediatric acute lymphoblastic leukemia. Hematol Oncol Clin North Am 23:973-90, v, 2009
