# Supplementary material for: Antileukemic Efficacy of Continuous vs Discontinuous Dexamethasone in Murine Models of Acute Lymphoblastic Leukemia
Source: PLoS One. 2015 Aug 7;10(8):e0135134. doi: 10.1371/journal.pone.0135134 (PMC4529108; doi:10.1371/journal.pone.0135134)
Supplement: S1 Table — All patients were treated on either the St. Jude Total XV or XVI protocols and remain in remission. Risk Group classification has been described previously for Total XV [48] and XVI [49] protocols. (DOCX) [file pone.0135134.s005.docx]

Supplement to Antileukemic efficacy of continuous vs discontinuous dexamethasone in murine models of acute lymphoblastic leukemia

Laura B. Ramsey^1^, Laura J. Janke^2^, Monique A. Payton^1^, Xiangjun Cai^1^, Steven W. Paugh^1^, Seth E. Karol^1^, Landry Kamdem Kamdem^3^, Cheng Cheng^4^, Richard T. Williams^5^, Sima Jeha^6^, Ching-Hon Pui^6^, William E. Evans^1^, Mary V. Relling^1*^

^1^Pharmaceutical Sciences Department, St. Jude Children’s Research Hospital, Memphis, TN, USA;

^2^Department of Pathology, St. Jude Children’s Research Hospital, Memphis, TN, USA;

^3^Harding University College of Pharmacy, Searcy, AR, USA;

^4^Biostatistics Department, St. Jude Children’s Research Hospital, Memphis, TN, USA;

^5^Puma Biotechnology Inc., Los Angeles, CA, USA;

^6^Department of Oncology, St. Jude Children’s Research Hospital, Memphis, TN, USA.

* Corresponding author:

Email: mary.relling@stjude.org (MVR)

**S1 Table. Patient demographics**

| Xenograft | Patient age (years) | Sex | ALL lineage | Subtype | Risk Group |
| --- | --- | --- | --- | --- | --- |
| SJTALL021916 | 18.6 | Male | T | T-ALL | Low |
| SJTALL033 | 8.0 | Male | T | T-ALL | Std/High |
| SJTALL030 | 8.9 | Male | T | T-ALL | Std/High |
| SJMLL005 | 18.7 | Female | B | MLL-AF4 | Std/High |
| SJBALL215 | 5.5 | Female | B | Early PreB | Std/High |
| SJE2A007 | 12.3 | Female | B | E2A-PBX1 | Std/High |
| SJMLL009 | 18.3 | Male | B | MLL-AF4 | Low |
| SJHYPO123 | 3.4 | Male | B | Hypodiploid | Std/High |

All patients were treated on either the St. Jude Total XV or XVI protocols and remain in remission. Risk Group classification has been described previously for Total XV [1] and XVI [2] protocols.

**References**

1. Pui CH, Campana D, Pei D, Bowman WP, Sandlund JT, Kaste SC, et al. Treating childhood acute lymphoblastic leukemia without cranial irradiation. The New England journal of medicine. 2009;360(26):2730-41.

2. Jeha S, Pui CH. Risk-adapted treatment of pediatric acute lymphoblastic leukemia. Hematology/oncology clinics of North America. 2009;23(5):973-90, v.
